# Musculoskeletal pathology as an early warning sign of systemic amyloidosis: a systematic review of amyloid deposition and orthopedic surgery

**DOI:** 10.1186/s12891-020-03912-z

**Published:** 2021-01-08

**Authors:** Austin E. Wininger, Brian M. Phelps, Jessica T. Le, Joshua D. Harris, Barry H. Trachtenberg, Shari R. Liberman

**Affiliations:** 1 Houston Methodist Orthopedics & Sports Medicine, 6445 Main Street, Outpatient Center, Suite 2500, Houston, TX 77030 USA; 2 Houston Methodist DeBakey Heart & Vascular Center, 6550 Fannin Street, Smith Tower, Suite 1901, Houston, TX 77030 USA

**Keywords:** Cardiac amyloidosis, Amyloid, Transthyretin, Immunoglobulin light-chain, Musculoskeletal soft tissue, Orthopedic surgery

## Abstract

**Background:**

Transthyretin and immunoglobulin light-chain amyloidoses cause amyloid deposition throughout various organ systems. Recent evidence suggests that soft tissue amyloid deposits may lead to orthopedic conditions before cardiac manifestations occur. Pharmacologic treatments reduce further amyloid deposits in these patients. Thus, early diagnosis improves long term survival.

**Questions/purposes:**

The primary purpose of this systematic review was to characterize the association between amyloid deposition and musculoskeletal pathology in patients with common orthopedic conditions. A secondary purpose was to determine the relationship between amyloid positive biopsy in musculoskeletal tissue and the eventual diagnosis of systemic amyloidosis.

**Methods:**

We performed a systematic review using PRISMA guidelines. Inclusion criteria were level I-IV evidence articles that analyzed light-chain or transthyretin amyloid deposits in common orthopedic surgeries. Study methodological quality, risk of bias, and recommendation strength were assessed using MINORS, ROBINS-I, and SORT.

**Results:**

This systematic review included 24 studies for final analysis (3606 subjects). Amyloid deposition was reported in five musculoskeletal pathologies, including carpal tunnel syndrome (transverse carpal ligament and flexor tenosynovium), hip and knee osteoarthritis (synovium and articular cartilage), lumbar spinal stenosis (ligamentum flavum), and rotator cuff tears (tendon). A majority of studies reported a mean age greater than 70 for patients with TTR or AL positive amyloid.

**Conclusions:**

This systematic review has shown the presence of amyloid deposition detected at the time of common orthopedic surgeries, especially in patients ≥70 years old. Subtyping of the amyloid has been shown to enable diagnosis of systemic light-chain or transthyretin amyloidosis prior to cardiac manifestations.

**Level of evidence:**

Level IV.

**Supplementary Information:**

The online version contains supplementary material available at 10.1186/s12891-020-03912-z.

## Introduction

Amyloidosis is a systemic disease characterized by extracellular deposition of misfolded protein fragments throughout the body. Amyloid proteins can deposit in any tissue or organ, and depending on the location, may lead to dysfunction due to compressive or degenerative pathology [1, 2]. Two of the most commonly misfolded protein precursors that lead to cardiac manifestations are transthyretin and immunoglobulin light-chain, accounting for > 95% of cardiac amyloidoses (Table 1). The systemic disease created by these two protein precursors are referred to as transthyretin amyloidosis (ATTR) and immunoglobulin light-chain amyloid amyloidosis (AL). ATTR has two subtypes: a wild type form (ATTRwt) and an inherited, mutant form (ATTRm) [3].

**Table 1:** Most common subtypes of systemic amyloidosis

Subtype of Systemic Amyloidosis	ATTRwt	ATTRm	AA	AL	AB2M
**Protein Deposited**	Transthyretin (wildtype)	Transthyretin (mutated)	Serum Amyloid A	Immunoglobulin light chain	Beta-2 Microglobulin
**Protein Function and Source**	Thyroxine and retinol binding protein made in liver and choroid plexus	Thyroxine and retinol binding protein made in liver and choroid plexus	Acute phase protein made in liver that accumulates with sustained and chronic inflammation	Immunoglobulin produced by clonal plasma cells in the bone marrow	Component of major histocompatibility complex type 1, which is on all nucleated cells
**Other Names for the Disease**	Senile restrictive cardiomyopathy	Familial cardiomyopathy, familial neuropathy	Secondary systemic amyloidosis	Primary systemic amyloidosis	Dialysis-related amyloidosis
**Major Organs Involved**	Heart, musculoskeletal, nervous	Heart, nervous, musculoskeletal	Kidney, nervous, heart, lung	Heart, kidney, liver, gastro-intestinal, nervous, lung, soft tissue	Autonomic nervous, musculoskeletal
**Implicated in Musculoskeletal Pathology**	Yes	Yes	Yes	Yes	Yes
**Treatment**	Novel therapies that decrease transthyretin production or stabilize transthyretin to prevent further amyloid deposition	Novel therapies that decrease transthyretin production or stabilize transthyretin to prevent further amyloid deposition	Treatment of underlying inflammatory condition	Chemotherapy directed at plasma cell clone	High flux hemodialysis membrane, renal transplantation

*ATTRwt* wild-type transthyretin amyloidosis, *ATTRm* mutant transthyretin amyloidosis, *AA* amyloid A amyloidosis, *AL* immunoglobulin light chain amyloidosis, *AB2M* beta-2 microglobulin amyloidosis

Both ATTR and AL result in varying degrees of extracellular amyloid deposits throughout the body, including the heart, nervous tissue, gastrointestinal tract, and musculoskeletal soft tissues [4, 5]. Cardiac amyloid deposition is the most predictive of morbidity and mortality due to a restrictive cardiomyopathy and subsequent heart failure [6, 7]. Median survival for ATTRwt and ATTRm with cardiac involvement is typically under 60 months [8, 9]. Once amyloid fibrils have deposited within the walls of the heart, it is irreversible. Recent pharmacologic advances for both ATTR and AL have resulted in treatments that can halt amyloid deposition, slow disease progression, decrease morbidity, and increase patient lifespan [9–11].

A significant proportion of patients with AL or ATTR develop amyloid deposition in musculoskeletal soft tissues, including ligaments, tendons, and articular cartilage [12–14]. Several studies have demonstrated that bilateral carpal tunnel syndrome often precedes the diagnosis of cardiac amyloidosis by 5 to 10 years [15–18]. A recent study revealed that 10% of males over 50 and females over 60 undergoing routine carpal tunnel release had amyloid positive tenosynovial biopsies [19]. Further workup revealed that two were found to have cardiac amyloidosis, one case of AL diagnosed by echocardiography and one case of ATTR by scintigraphy [19]. The prevalence of other musculoskeletal pathologies in the setting of multiorgan amyloidosis has not been well described in the literature. Recent data suggests that in addition to carpal tunnel syndrome, lumbar spinal stenosis may be an early manifestation of cardiac amyloidosis due to amyloid deposition in the ligamentum flavum [20, 21]. Moreover, TTR and light-chain amyloid deposits have been reported in synovial tissue obtained during hip and knee arthroplasties, with one study revealing that patients with ATTR cardiomyopathy were over five times more likely to have undergone total hip arthroplasty than the general population [22–27]. A focused review on the musculoskeletal manifestations of amyloidosis suggested a possible role of histological screening for amyloidosis during common orthopedic surgeries [28]. Current literature is lacking a systematic review on the results of biopsy samples of musculoskeletal soft tissue in regard to the presence of amyloid and the eventual diagnosis of systemic amyloidosis.

The primary purpose of this systematic review was to characterize the association between amyloid protein deposition and musculoskeletal pathology in patients undergoing common orthopedic surgeries. A secondary purpose was to determine the relationship between amyloid presence detected in musculoskeletal tissue at the time of orthopedic surgery and the eventual diagnosis of systemic amyloidosis. The authors hypothesize that both TTR and immunoglobulin light-chain amyloid would be found in musculoskeletal soft tissue biopsy of patients undergoing orthopedic surgeries and that a small percentage of these patients would eventually be diagnosed with systemic amyloidosis.

## Methods

A systematic review was performed following Preferred Reporting Items for Systematic Reviews and Meta-analyses (PRISMA) guidelines [29]. The authors of this study conducted separate searches of the following databases since their inceptions to present day: PubMed, Scopus, Web of Science, and Google Scholar on March 30, 2020. The main search was performed in PubMed using controlled vocabulary (MeSH) and natural language (title, abstract, and other terms). Search terms focused on (1) amyloid, amyloid deposits, amyloidosis and (2) orthopedics, orthopedic disorders, and musculoskeletal disease (Additional Table 1). These terms were tested for relevancy in PubMed and once finalized, were translated into Scopus, Web of Science, and Google Scholar for article retrieval. All article duplicates were removed prior to screening.

Eligible studies consisted of Level I-IV studies published in the English language prior to March 30, 2020, that investigated light-chain or TTR amyloid deposits in musculoskeletal tissues of patients being treated for common orthopedic conditions. Both print and electronically published journal articles were eligible for inclusion. Screening was performed independently by two reviewers (AEW and BMP) using a prior methodology following PRISMA guidelines (Additional Table 2) [30].

Initial screening of titles and abstracts were performed in Rayyan QCRI using the predetermined inclusion criteria on whether the manuscript discussed the presence of amyloid in tissue samples removed during routine musculoskeletal operations. After initial screening, full-text articles were assessed for eligibility. Any disagreements at the end of each step were settled by discussion between the two reviewers. In all cases, a consensus was reached. All references within included studies were cross-referenced and assessed for potential inclusion if missed by the initial search. Studies that were not amyloid focused or that focused on secondary causes of amyloidosis, such as beta-2 microglobulin amyloid related to dialysis or serum amyloid A related to chronic inflammatory conditions, were excluded. Studies that evaluated uncommon orthopedic conditions, such as multiple myeloma, soft-tissue or osseous amyloidoma, and proximal myopathy, were also excluded. Level V evidence expert opinion, case reports, and letters to editors were excluded. Medical conference abstracts and synthetic review articles (systematic review, meta-analysis, scoping review) were excluded as well. Duplicate subject publications within separate unique studies were not reported more than once. In the situation of duplicate studies from the same author(s) and/or institution(s) reporting on the same or overlapping subjects, only one study was retained (either highest level of evidence, largest number of subjects, longest follow-up, or most pertinent primary outcome score [more explicit reporting of histopathological amyloid findings or with a more thorough examination for concomitant systemic amyloidosis] [or relevant secondary outcome score {s}]) and the other(s) were excluded.

From each article, details regarding the participants (male/female, mean age, diagnosis) and interventions (surgery, biopsy type, biopsy location, and presence of TTR or light-chain amyloid positive biopsy), outcomes (subjective patient-reported outcomes, objective clinician measured outcomes [motion, strength, mortality/survival]). Study type and design were assessed. Levels I, II, III, and IV of evidence were assigned to studies according to the Oxford Centre for Evidence Based Medicine used by the American version of the Journal of Bone and Joint Surgery [31]. Study-specific demographics, interventions, and biopsy data were extracted from each study.

The methodological quality of the included studies was assessed using the MINORS (methodological index for non-randomized studies) criteria [32]. The risk of bias was evaluated using the ROBINS-I (Risk of Bias in Non-randomized Studies-of Interventions) which assigns an overall bias as low, moderate, severe or critical [33]. Recommendation regarding the quality, quantity, and consistency of evidence was made using SORT (Strength of Recommendation Taxonomy) [34]. These assessments were performed by one author (AEW) and independently checked by another author (BMP).

## Results

**Fig. 1:**
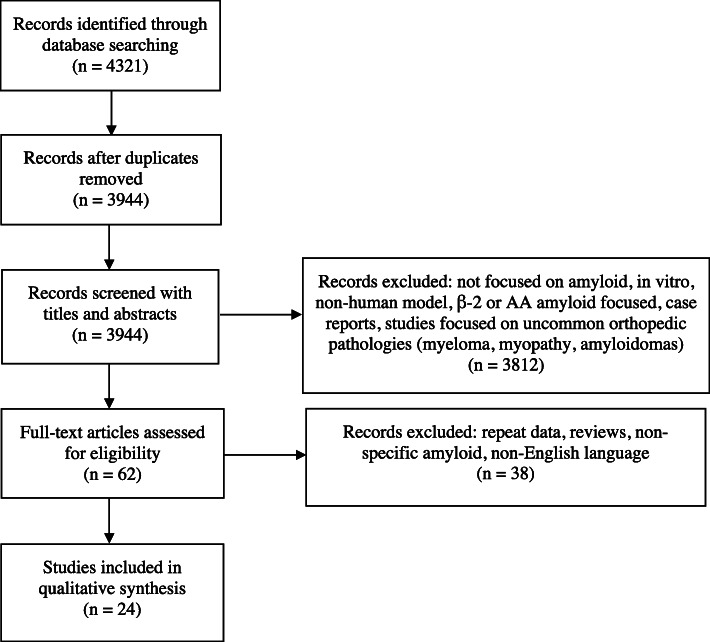
Flow chart application of exclusion criteria for study inclusion

Database searches of PubMed, Scopus, Web of Science, and Google Scholar resulted in 3944 unique abstracts. Of the 3944 abstracts screened, 3812 did not meet our inclusion criteria. After screening, 62 full-text articles were assessed for eligibility, with 24 articles included in the final analysis (Fig. 1, Table 2).

**Table 2:** Summary of key elements of assessed studies

Authors	Year	Journal	Country	Level of Evidence	Study type	Study Design	Length of Follow Up (months)
Akasaki et al. [35]	2015	Arthritis Rheumatol	United States (California)	3	Diagnostic	Case control	none
aus dem Siepen et al. [36]	2019	Clin Res Cardiol	Germany	3	Diagnostic	Retrospective comparative	29 ± 29
Bishop et al. [15]	2018	Amyloid	United States (Maryland)	3	Diagnostic	Retrospective comparative	none
Fernandez Fuertes et al. [37]	2017	Med Clin (Barc)	Spain	3	Diagnostic	Prospective cohort*	36
Geller et al. [38]	2017	JAMA	United States (Massachusetts)	3	Diagnostic	Case control	none
Gies et al. [39]	1996	Clin Neuropathol	Germany	4	Diagnostic	Case series	none
Gioeva et al. [14]	2013	Amyloid	Germany	4	Diagnostic	Case series	none
Gu et al. [23]	2014	J Zhejiang Univ Sci B	China	3	Diagnostic	Case control	none
Kyle et al. [40]	1992	Am J Clin Pathol	United States (Minnesota)	4	Diagnostic	Case series	168
Nakamichi et al. [41]	1996	Muscle Nerve	Japan	4	Diagnostic	Case series	56
Niggemeyer et al. [42]	2011	Arch Orthop Trauma Surg	Germany	4	Diagnostic	Case series	none
Rubin et al. [27]	2017	Amyloid	United States (New York)	3	Diagnostic	Retrospective comparative	none
Samões et al. [43]	2017	Amyloid	Portugal	4	Diagnostic	Case series	38.4
Scott et al. [44]	2019	Plast Reconstr Surg	United States (Arizona)	4	Diagnostic	Case series	none
Sekijima et al.18	2011	Hum Pathol	Japan	3	Diagnostic	Case control	none
Sperry et al. [45]	2018	J Am Coll Cardiol	United States (Ohio)	2	Diagnostic	Prospective cohort	next phase of study
Stein et al. [46]	1987	Virchows Arch A Pathol Anat Histopathol	Germany	4	Diagnostic	Case series	none
Sueyoshi et al. [13]	2011	Hum Pathol	Japan	4	Diagnostic	Case series	none
Takanashi et al. [25]	2013	Amyloid	Japan	4	Diagnostic	Case series	none
Uchihara et al. [47]	2018	Pathol Res Pract	Japan	4	Diagnostic	Case series	none
Westermark et al. [20]	2014	Ups J Med Sci	Sweden	4	Diagnostic	Case series	none
Yanagisawa et al. [21]	2015	Mod Pathol	Japan	4	Diagnostic	Case series	none
Yanagisawa et al. [26]	2016	Amyloid	Japan	4	Diagnostic	Case series	none
Zegri-Reiriz et al. [48]	2019	J Cardiovasc Transl Res	Spain	3	Diagnostic	Prospective cohort^a^	none

^a^no consistently applied reference standard

The 24 studies in this systematic review included a total of 13 cohorts of patients with carpal tunnel syndrome, six with lumbar spinal stenosis, five with knee osteoarthritis, two with hip osteoarthritis, two with rotator cuff pathology, and two with biceps tendon pathology. Nineteen of the included studies reported results from musculoskeletal biopsy samples: 10 from transverse carpal ligament or flexor tenosynovium during carpal tunnel release, four from ligamentum flavum during lumbar decompression, four from synovium and cartilage during total knee arthroplasty, one from synovium and cartilage during total hip arthroplasty, and one from rotator cuff tendon during rotator cuff repair (Table 3).

**Table 3:** Summary of key data and findings of assessed studies

Authors	Total Subjects	Male/ Female	Mean Age (years)	Orthopedic Pathology	Biopsy location	Key Findings
Akasaki et al. [35]	36	16/20	60.8	knee OA	cartilage	Young normal cartilage: 0% amyloid deposits, 0% TTR detected; Aged normal cartilage: 58% amyloid deposits, 83% TTR detected; OA cartilage: 100% amyloid deposits, 100% TTR detected
aus dem Siepen et al. [36]	466	373/93	63.7	CTS and LSS	none	Latency between CTR and diagnosis of systemic amyloidosis was significantly longer in ATTRwt compared to ATTRm (117 ± 179 months vs 66 ± 73 months; *p*=0.02)
Bishop et al. [15]	82	55/27	70.5	CTS	none	CTS associated with a longer delay in diagnosis (OR: 2.13; 95% CI 1.49–3.03) in 82 patients with AL or ATTR cardiomyopathy
Fernandez Fuertes et al. [37]	147	31/116	58	CTS	TCL, FTS	29 of 147 (19.7%) of patients undergoing CTR had amyloid positive biopsies; 4 patients later developed systemic amyloidosis
Geller et al. [38]	151	137/14	74.7	Distal biceps rupture	none	33.3% [95% CI, 24.7–42.9%] of 111 patients with ATTRwt cardiomyopathy had ruptured distal biceps tendon on exam
Gies et al. [39]	100	60/40	NS	LSS	LF	12 of 100 specimens from LSS and LDH patients contained amyloid; 5 contained very strong TTR presence
Gioeva et al. [14]	1020	351/669	61.8	CTS	TCL	98 biopsies contained TTR amyloid; 70 of 81 patients with DNA sequencing had wildtype TTR gene
Gu et al. [23]	36	16/20	66.4	knee OA	knee synovium	9 of 36 knee OA patients had amyloid positive biopsies; 8 contained TTR amyloid and 1 contained light-chain amyloid
Kyle et al. [40]	35	23/12	71	CTS	TCL, FTS	33 of 35 had TTR amyloid; 2 developed systemic amyloidosis
Nakamichi et al. [41]	108	5/103	56	CTS	TCL, FTS	10 of 108 patients had amyloid deposits, 6 contained TTR amyloid
Niggemeyer et al. [42]	50	14/36	68.4	Hip OA	synovium, cartilage	17 of 50 consecutive patients had amyloid deposits, all contained TTR amyloid
Rubin et al. [27]	313	234/79	66.6	knee and hip OA, rotator cuff	none	THA and TKA significantly more common among ATTR patients with cardiomyopathy (THA: RR 5.61, 95% CI 2.25–4.64; TKA: RR 3.32, 95% CI 2.25–4.64)
Samões et al. [43]	16	3/13	46.1	CTS	TCL	In 16 patients with known ATTRm, 15 patients had CTS that preceded amyloidosis and 14 had amyloid positive biopsies
Scott et al. [44]	35	16/19	72	recurrent CTS	FTS	9 of 35 patients (26%) with recurrent CTS had an amyloid positive biopsy, 7 of which contained TTR
Sekijima et al. [18]	132	40/92	71.8	CTS	FTS	34 of 100 patients with idiopathic CTS had TTR amyloid deposits versus 7 of 32 autopsy controls (OR, 15.8; 95% CI, 3.3–75.7)
Sperry et al. [45]	98	51/47	68.5	CTS	FTS	10 of 98 patients undergoing CTR had an amyloid positive biopsy (7 TTR, 2 light chain); 3 diagnosed with systemic amyloidosis
Stein et al. [46]	108	NS	NS	CTS	TCL	Amyloid deposits were found in 23 of 108 (21%) patients with idiopathic CTS; TTR was identified in 14 of these 23 patients
Sueyoshi et al. [13]	111	56/55	62	CTS, LSS, rotator cuff	FTS, LF, RCT	39 of 111 specimens contained TTR amyloid deposits
Takanashi et al. [25]	232	31/201	73	knee OA	synovium	21 of 232 knee OA patients (9%) had TTR amyloid deposits
Uchihara et al. [47]	25	NS	NS	NS	periarticular F&A	3 of 25 specimens contained TTR amyloid
Westermark et al. [20]	26	13/13	66.5	LSS	LF, bone fragments	21 of 26 specimens contained amyloid deposits; 5 of 15 specimens suitable for immunohistochemistry contained TTR amyloid
Yanagisawa et al. [21]	95	68/27	70.7	LSS	LF	All 95 LF specimens resected from LSS patients contained amyloid deposits; 43 contained TTR amyloid
Yanagisawa et al. [26]	52	17/35	66.6	knee OA	periarticular knee	TTR amyloid deposits were found in specimens from: 18 of 51 menisci, 8 of 27 articular cartilage, and 6 of 34 synovium
Zegri-Reiriz et al. [48]	101	32/69	69	CTS	none, myocardial	Prevalence of cardiac amyloidosis in the cohort was 1.2% (3/233) and 5.5% (3/55) for patients with LVH and bilateral CTS

*CI* confidence interval, *CTS* carpal tunnel syndrome, *DNA* deoxyribonucleic acid, *F&A* foot and ankle, *FTS* flexor tenosynovium, *LF* ligamentum flavum, *LDH* lumbar disc herniation, *LSS* lumbar spinal stenosis, *LVH* left ventricular hypertrophy, *NS* not specified, *OA* osteoarthritis, *OR* odds ratio, *RCT* rotator cuff tendon, *RR* relative risk, *TCL* transverse carpal ligament, *THA* total hip arthroplasty, *TKA* total knee arthroplasty

Immunohistochemical testing was completed to subtype the amyloid with report on the occurrence of TTR or light-chain amyloid deposits (Additional Table 3). Overall, 3606 patients were included across all studies (1625 males, 1956 females). Of these, 2183 patients underwent biopsy of musculoskeletal soft tissue, with 410 TTR positive biopsies and 8 light-chain positive biopsies. Among the 1753 patients (520 males, 1071 females) who underwent carpal tunnel releases with biopsy. TTR positive biopsies were identified in 241 of these patients and 7 had light-chain positive biopsies. There were 157 patients (81 males, 40 females) who underwent lumbar decompression for spinal stenosis with biopsy. TTR positive biopsies were identified in 64 of these patients and 0 had light-chain positive biopsies. Among the 382 patients (82 males, 294 females) who underwent hip or knee arthroplasty with biopsy, TTR positive biopsies were identified in 97 of these patients and 1 had light-chain positive biopsies (Table 4). A majority of studies reported a mean age greater than 70 for patients with TTR or AL positive amyloid. To diagnose either ATTR or AL after positive biopsy, cardiac workup with electrocardiogram, echocardiography, or scintigraphy and hematology workup with urine and serum monoclonal antibody tests could be considered.

**Table 4:** The incidence of TTR and immunoglobulin light-chain amyloid from tissue samples removed during common orthopedic operations

	Total Patients	Male/Female (when specified)	Patients with Orthopedic Biopsy	TTR + Biopsy	Ig Light-Chain + Biopsy	% of patients with TTR or light-chain+ Biopsy
**All Studies**	3606	1625/1956	2183	410	8	19.1%
**CTS with Biopsy**	1753	520/1071	1753	241	7	14.1%
**LSS with Biopsy**^**a**^	157	81/40	157	64	0	40.8%
**Hip and Knee OA with Biopsy**	382	82/294	382	97	1	25.7%

*CTS* carpal tunnel syndrome, *Ig* immunoglobulin, *LSS* lumbar spinal stenosis, *%* percentage, *+* positive, *OA* osteoarthritis

^a^Excludes Gies et al. study due to the authors not differentiating lumbar spinal stenosis patients from lumbar disc herniation patients

An attempt was made to extract outcomes data from each study, however patient follow-up was poorly reported. Of the patients with amyloid positive amyloid biopsies collected at the time of carpal tunnel release, nine were subsequently diagnosed with systemic amyloidosis (Table 5). Systemic amyloidosis was diagnosed by genetic testing, a second biopsy location, physical examination, electrocardiography, N-terminal pro–B-type natriuretic peptide, troponin T, echocardiography, or technetium pyrophosphate nuclear scintigraphy. No diagnosis of systemic or cardiac amyloidosis was reported in patients with positive biopsies undergoing lumbar decompression, hip, or knee arthroplasty.

**Table 5:** Results of studies reporting on biopsies taken during carpal tunnel release of seemingly idiopathic carpal tunnel syndrome

Study	Patients with Biopsy	Amyloid Positive Biopsy	Follow Up	Diagnosed with Systemic Amyloidosis	Type of Systemic Amyloidosis
Fernandez Fuertes et al. [37]	147	29	36 months	4	3 AL, 1 ATTR
Kyle et al. [40]	35	35	11 years	2	1 ATTR, 1 untyped
Gioeva et al. [14]	1010	98	None	NR	
Nakamichi et al. [41]	108	10	12.5 years	0	
Sekijima et al. [18]	100	34	None	NR	
Sperry et al. [45]	98	10	None, concomitant workup for systemic disease if biopsy positive	3	2 ATTR, 1 AL
Stein et al. [46]	108	19	None	NR	
Sueyoshi et al. [13]	54	20	None	NR	

Study methodological quality was assessed using MINORS and the risk of bias using ROBINS-I (Table 6). Although these studies indicate that orthopedic pathology can be an early warning sign or early manifestation of cardiac amyloidosis, most only report the histological findings and are of very low methodological quality with critical or serious risk of bias. Twenty of the 24 studies do not include long term follow up or monitor for current or future cardiac manifestations of amyloidosis. As a body of evidence, the included studies are SORT C, indicating the recommendation level is based on consensus, usual practice, opinion, or disease-oriented evidence.

**Table 6:** Critical appraisal of included studies using MINORS and ROBINS-I

Study	MINORS (non-comparative study, out of 16)	MINORS (comparative study, out of 24)	ROBINS-I (across all domains)
Akasaki et al. [35]		12	serious
aus dem Siepen et al. [36]	7		critical
Bishop et al. [15]	6		critical
Fernandez Fuertes et al. [37]	11		critical
Geller et al. [38]		12	critical
Gies et al. [39]	8		critical
Gioeva et al. [14]		6	critical
Gu et al. [23]		12	serious
Kyle et al. [40]	8		moderate
Nakamichi et al. [41]	10		serious
Niggemeyer et al. [42]	8		critical
Rubin et al. [27]		10	serious
Samões et al. [43]	9		moderate
Scott et al. [44]	4		critical
Sekijima et al. [18]		15	serious
Sperry et al. [45]	10		moderate
Stein et al. [46]	6		critical
Sueyoshi et al. [13]	5		critical
Takanashi et al. [25]	8		moderate
Uchihara et al. [47]	2		critical
Westermark et al. [20]	6		serious
Yanagisawa et al. [21]	4		serious
Yanagisawa et al. [26]	6		serious
Zegri-Reiriz et al. [48]	8		moderate

## Discussion

This systematic review supports the author’s hypotheses that amyloid deposits are present within musculoskeletal soft tissues encountered during common orthopedic surgeries and identifying their presence can aid in diagnosing ATTR or AL. This systematic review also supports that histological testing of orthopedic biopsy samples may enable screening for unsuspected systemic amyloidosis. Despite the heterogeneity of included studies, the reported findings suggest that ATTR and AL can lead to amyloid deposits that are present in locations of musculoskeletal pathology. TTR amyloid was much more commonly observed than light-chain amyloid within orthopedic biopsies (Table 4).

For carpal tunnel syndrome, the data from Sperry et al. lead Donnelly et al. to propose an algorithm for treating patients undergoing carpal tunnel release [19, 45]. In this, men over the age of 50 and women over the age of 60 with bilateral symptoms or prior release are designated to be in tier 1. History of spinal stenosis, biceps tendon rupture, atrial fibrillation/flutter, pacemaker, heart failure, or family history of amyloidosis are tier 2. If a patient meets the criteria for tier 1 and has one or more risk factors listed in tier 2, the algorithm recommends a biopsy, amyloid subtyping, and referral to amyloid specialist if found to be positive for TTR or light-chain. Patients presenting with recurrent carpal tunnel syndrome in the setting of true idiopathic disease with no obvious risk factors may be at higher risk for having amyloid deposits within their transverse carpal ligament and flexor tenosynovium [44]. This amyloid was subtyped as TTR deposits in several patient cohorts, but no true prospective follow-up for the development of cardiac amyloidosis has been performed.

Several studies report an association between lumbar spinal stenosis and TTR amyloid deposits within the ligamentum flavum. Both thickened ligamentum flavum and increased lumbar spine instability were associated with a greater quantity of TTR amyloid deposits in spinal stenosis patients [21]. Elderly men with spinal stenosis have been labeled as the most at risk for ATTR and with current evidence may warrant a biopsy [20, 49]. In patients undergoing surgery for lumbar disc herniation, with no additional risk factors for amyloidosis, the literature indicates that amyloid deposition is most often absent from biopsy tissues [21, 39].

Studies on hip and knee arthroplasty related to cardiac amyloidosis have indicated the presence of TTR and light-chain amyloid in hip and knee synovium. Whether the amyloid deposition in the synovium is age associated and found by coincidence, or corresponds to a diagnosis of systemic disease, has not been elucidated [27, 42].

Regarding other orthopedic pathology, one study revealed a high occurrence of distal biceps tendon rupture in patients diagnosed with ATTRwt cardiomyopathy [38]. A study reporting the results of rotator cuff biopsies did not provide information beyond the presence of TTR amyloid [13]. In a similar manner, the only foot and ankle study reporting on the presence of TTR amyloid, did not provide any clinical correlation with the positive biopsies [47]. Without a history of carpal tunnel syndrome or lumbar spinal stenosis, biopsy may not be warranted with these pathologies unless there is a red flag in the patient’s medical history.

Systemic amyloidosis may be more common than previously recognized [50, 51] and current pharmacologic options do not reverse amyloid deposition [52]. For ATTR, three treatments recently received FDA approval, and for AL, improvements in chemotherapy have markedly improved survival, even in cases with cardiac involvement [1, 53, 54]. ATTR and AL are difficult diseases to diagnose due to nonspecific symptoms, overlapping diagnoses, and under-recognition by physicians. As patients could potentially present with musculoskeletal conditions prior to systemic manifestations, orthopedic surgeons may play a role in early diagnosis.

## Limitations

Limitations of this systematic review include predominantly retrospective level-III/IV evidence included for review. The heterogeneous and limited data on gender, age, clinical outcomes, or histopathological findings were unable to be quantitatively assimilated, precluding meta-analysis. Current studies contain little or no follow-up information or monitoring of patients for development of systemic disease or restrictive cardiomyopathy. Moreover, few studies utilized genetic testing and no studies evaluated for serum testing that could rule in or out the need for biopsy pre-operatively. There is a lack of data regarding various musculoskeletal soft tissues of common orthopedic pathology, such as Achilles tendon, patellar tendon, and hip labrum. There is also a lack of data regarding osteoarthritic joints other than the hip and knee. The limitations of any systematic review are a result of the studies they include and analyze. As such, the above-mentioned shortcomings may limit the fidelity of clinical relevance.

## Future research directions

There have been no prospective studies that evaluate all patients regardless of biopsy results for underlying amyloidosis. The results of such a study would enable a more robust analysis of the diagnostic potential of orthopedic biopsies for cardiac amyloidosis. No studies have evaluated the economic implications of a musculoskeletal soft tissue biopsy at the time of orthopedic procedure, but prior authors have indicated that the low cost of screening may avoid the expense of treating progressive heart failure [19]. Future studies should look at the economic impact of a larger testing protocol.

## Conclusion

This systematic review supports the presence of amyloid deposition detected at the time of common orthopedic surgeries, most commonly in patients ≥70 years old. Subtyping of the amyloid can enable diagnosis of light-chain or TTR amyloidosis prior to cardiac manifestations. Patient characteristics that may lead orthopedic surgeons to consider the potential need for amyloid biopsy include: a family member with amyloidosis, a personal history of unexplained peripheral neuropathy or autonomic dysfunction, atrial fibrillation, heart failure, or pacemaker with no definitive cause, or an orthopedic history involving bilateral carpal tunnel syndrome, lumbar spinal stenosis, or multiple atraumatic tendon ruptures. Further prospective studies are needed to better determine when and in what musculoskeletal tissues there may be clinical benefit of biopsy for amyloidosis.

## Supplementary Information

**Additional file 1.**

**Additional file 2.**

**Additional file 3.**

## Data Availability

All data generated or analyzed during this study are included in this published article.

## Abbreviations

AL: Immunoglobulin light-chain amyloid amyloidosis
ATTR: Transthyretin amyloidosis
ATTRm: Mutant transthyretin amyloidosis
ATTRwt: Wild type transthyretin amyloidosis
MeSH: Medical subject headings
MINORS: Methodological index for non-randomized studies
PRISMA: Preferred reporting items for systematic reviews and meta-analyses
ROBINS-I: Risk of bias in non-randomized studies-of interventions
SORT: Strength of recommendation taxonomy
TTR: Transthyretin
