# Supplementary material for: Musculoskeletal pathology as an early warning sign of systemic amyloidosis: a systematic review of amyloid deposition and orthopedic surgery
Source: BMC Musculoskelet Disord. 2021 Jan 8;22:51. doi: 10.1186/s12891-020-03912-z (PMC7796584; doi:10.1186/s12891-020-03912-z)
Supplement: Supplementary file 1 — Additional file 1. [file 12891_2020_3912_MOESM1_ESM.docx]

**"Amyloid"[Mesh] OR "Amyloidosis"[Mesh]**

amyloid OR

amyloidosis OR

"Amyloid Deposits" OR

"Amyloid Deposit" OR

"Amyloid Plaques" OR

"amylois substance" OR

"amyloid fibrils" OR

transthyretin OR

apolipoprotein OR

"senile systemic amyloidosis"

(("Amyloid"[Mesh] OR "Amyloidosis"[Mesh]) OR (amyloid[Other Term] OR amyloidosis[Other Term] OR "Amyloid Deposits"[Other Term] OR "Amyloid Deposit"[Other Term] OR "Amyloid Plaques"[Other Term] OR "amyloid fibrils"[Other Term] OR transthyretin[Other Term] OR apolipoprotein[Other Term] OR "senile systemic amyloidosis"[Other Term])) OR (amyloid[Text Word] OR amyloidosis[Text Word] OR "Amyloid Deposits"[Text Word] OR "Amyloid Deposit"[Text Word] OR "Amyloid Plaques"[Text Word] OR "amyloid fibrils"[Text Word] OR transthyretin[Text Word] OR apolipoprotein[Text Word] OR "senile systemic amyloidosis"[Text Word])

**"Orthopedics"[Mesh] OR "Musculoskeletal Diseases"[Mesh]**

"Musculoskeletal Disease" OR

"Orthopedic Disorders" OR

"Orthopedic Disorder" OR

"tendon injury" OR

"tendon injuries" OR

tendinopathy OR

tendinitis OR

tenosynovitis OR

osteoarthritis OR

"spinal stenosis" OR

"spinal canal stenosis" OR

"carpal tunnel syndrome"

(("Orthopedics"[Mesh] OR "Musculoskeletal Diseases" [Mesh]) OR ("Musculoskeletal Disease" [Other Term] OR "Orthopedic Disorders" [Other Term] OR "tendon injury"[Other Term] OR "tendon injuries"[Other Term] OR tendinopathy [Other Term] OR tendinitis [Other Term] OR tenosynovitis [Other Term] OR osteoarthritis [Other Term] OR "spinal stenosis" [Other Term] OR "spinal canal stenosis"[Other Term] OR "carpal tunnel syndrome" [Other Term])) OR ("Musculoskeletal Disease" [Text Word] OR "Orthopedic Disorders"[Text Word] OR "Orthopedic Disorder"[Text Word] OR "tendon injury" [Text Word] OR "tendon injuries"[Text Word] OR tendinopathy[Text Word] OR tendinitis[Text Word] OR tenosynovitis[Text Word] OR osteoarthritis[Text Word] OR "spinal stenosis" [Text Word] OR "spinal canal stenosis" [Text Word] OR "carpal tunnel syndrome"[Text Word])

**"Tissues"[Mesh]**

tissue OR

ligament OR

ligaments OR

tendon OR

tendons OR

tenosynovium OR

synovium OR

"synovial tissue" OR

"synovial membrane" OR

capsule OR

"ligamentum flavum"

("Tissues"[Mesh] OR (tissue[Other Term] OR ligament[Other Term] OR ligaments[Other Term] OR tendon[Other Term] OR tendons[Other Term] OR tenosynovium[Other Term] OR synovium[Other Term] OR "synovial tissue"[Other Term] OR "synovial membrane"[Other Term] OR capsule[Other Term] OR "ligamentum flavum"[Other Term])) OR (tissue[Text Word] OR ligament[Text Word] OR ligaments[Text Word] OR tendon[Text Word] OR tendons[Text Word] OR tenosynovium[Text Word] OR synovium[Text Word] OR "synovial tissue"[Text Word] OR "synovial membrane"[Text Word] OR capsule[Text Word] OR "ligamentum flavum"[Text Word])
