# Supplementary material for: Musculoskeletal pathology as an early warning sign of systemic amyloidosis: a systematic review of amyloid deposition and orthopedic surgery
Source: BMC Musculoskelet Disord. 2021 Jan 8;22:51. doi: 10.1186/s12891-020-03912-z (PMC7796584; doi:10.1186/s12891-020-03912-z)
Supplement: Supplementary file 3 — Additional file 3. [file 12891_2020_3912_MOESM3_ESM.docx]

| **Authors** | **Total Patients** | **Patients in Group** | **Patient Characteristics** | **Orthopedic Diagnosis (n)** | | | | | | **Male/ Female** | **Average Age (SD or range)** | **Intervention** | **Biopsy Sample** | **TTR + Biopsy** | **Ig Light Chain + Biopsy** |
| --- | --- | --- | --- | --- | --- | --- | --- | --- | --- | --- | --- | --- | --- | --- | --- |
|  |  |  |  | **CTS** | **Hip OA** | **Knee OA** | **LSS** | **Rotator Cuff** | **Biceps Tendon** |  |  |  |  |  |  |
| Akasaki et al^48^ | 36 | 12 | young controls |  |  |  |  |  |  | 8/4 | 31 (9.1) | autopsy | knee joint cartilage | 0 |  |
|  |  | 12 | aged controls |  |  | 12 |  |  |  | 4/8 | 75.9 (6.3) | autopsy |  | 7 |  |
|  |  | 12 | undergoing TKA |  |  | 12 |  |  |  | 4/8 | 75.6 (13.1) | TKA |  | 12 |  |
| aus dem Siepen et al^49^ | 466 | 253 | ATTRwt | 152 |  |  | 36 |  |  | 232/21 | 74 (6) | retrospective chart review | no biopsy | | |
|  |  | 136 | ATTRm | 77 |  |  | 7 |  |  | 124/12 | 59 (12) |  |  |  |  |
|  |  | 77 | ATTRm gene carrier | 10 |  |  | 0 |  |  | 17/60 | 38 (11) |  |  |  |  |
| Bishop et al^15^ | 82 | 52 | ATTRwt and ATTRm | 27 |  |  |  |  |  | 42/10 | 73.4 (9.2) | retrospective chart review | no biopsy | | |
|  |  | 30 | AL | 6 |  |  |  |  |  | 13/17 | 65.6 (9) |  |  |  |  |
| Fernandez Fuertes et al^50^ | 147 | 147 | idiopathic CTS | 147 |  |  |  |  |  | 31/116 | 58 (14) | CTR | TCL, FTS |  | 3 |
| Geller et al^40^ | 151 | 111 | ATTRwt with HF |  |  |  |  |  | 37 | 108/3 | 74.9 (6) | resisted elbow flexion | no biopsy | | |
|  |  | 40 | HF, no amyloidosis |  |  |  |  |  | 1 | 29/11 | 74.1 (8) |  |  |  |  |
| Gies et al^38^ | 100 | 100 | total population |  |  |  | 14 |  |  | 60/40 | (22-82) | lumbar decompression | LF |  |  |
|  |  | 5 | LDH or LSS TTR + |  |  |  | NS |  |  | 1/4 | 69.8 (51-82) |  |  | 5 |  |
| Gioeva et al^14^ | 1020 | 25 | TTR + biopsy | 98 |  |  |  |  |  | 10/15 | 79 (59-87) | CTR | TCL | 98 |  |
|  |  | 73 | TTR + biopsy |  |  |  |  |  |  | 37/36 | 79 (59-92) |  |  |  |  |
|  |  | 922 | TTR - biopsy | 922 |  |  |  |  |  | 304/618 | 60 (17-93) |  |  |  |  |
| Gu et al^23^ | 36 | 28 | TTR - biopsy |  |  | 36 |  |  |  | 13/15 | 65.5 (58-72) | TKA | synovium |  | 1 |
|  |  | 8 | TTR + biopsy |  |  |  |  |  |  | 3/5 | 69.5 (65-72) |  |  | 8 |  |
| Kyle et al^51^ | 35 | 35 | amyloid + biopsy | 35 |  |  |  |  |  | 23/12 | 71 (53-93) | CTR | TCL, FTS | 33 |  |
| Nakamichi et al^52^ | 108 | 108 | idiopathic CTS | 108 |  |  |  |  |  | 5/103 | 56 (45-85) | CTR | TCL, FTS | 6 |  |
| Niggemeyer et al^39^ | 50 | 50 | total population |  | 50 |  |  |  |  | 14/36 | 68.4 (7.6) | THA | synovium, cartilage, bone |  |  |
|  |  | 17 | TTR + biopsy |  |  |  |  |  |  |  | 73.8 (60-87) |  |  | 17 |  |
| Rubin et al^27^ | 313 | 108 | ATTRwt with cardiomyopathy |  | 15 THA | 20 TKA |  | 14 RCR |  | 100/8 | 75 (7) | retrospective chart review - had patient undergone THA or TKA? | no biopsy | | |
|  |  | 64 | ATTRm with cardiomyopathy |  | 7 THA | 5 TKA |  | 3 RCR |  | 45/19 | 67 (10) |  |  |  |  |
|  |  | 141 | AL with cardiomyopathy |  | 6 THA | 9 TKA |  | 7 RCR |  | 89/52 | 60 (11) |  |  |  |  |
| Samões et al^53^ | 16 | 16 | TTR V30M mutation | 16 |  |  |  |  |  | 3/13 | 46.1 (30-70) | CTR | TCL | 14 |  |
| Scott et al^36^ | 35 | 35 | revision CTR | 35 |  |  |  |  |  | 16/19 | 72 (52-88) | revision CTR | FTS | 7 | 1 |
| Sekijima et al^18^ | 132 | 66 | idiopathic CTS | 100 |  |  |  |  |  | 9/57 | 63.3 (11.5) | CTR | FTS |  |  |
|  |  | 34 | idiopathic CTS |  |  |  |  |  |  | 17/17 | 75.1 (8.9) |  |  | 34 |  |
|  |  | 32 | autopsy control |  |  |  |  |  |  | 14/18 | 85.8 (8.4) | carpal tunnel dissection |  | 7 |  |
|  |  |  | autopsy control |  |  |  |  |  |  |  |  |  |  |  |  |
| Sperry et al^35^ | 98 | 88 | amyloid - biopsy | 98 |  |  |  |  |  | 45/43 | 68 (60-74) | CTR | FTS |  |  |
|  |  | 8 | TTR + biopsy |  |  |  | 4 |  | 1 | 6/4 | 72.5 (65-80) |  |  | 8 |  |
|  |  | 2 | Ig light chain + biopsy |  |  |  | 2 |  | 1 |  |  |  |  |  | 2 |
| Stein et al^54^ | 108 | 79 | amyloid - biopsy | 108 |  |  |  |  |  | NS | NS | CTR | TCL |  |  |
|  |  | 29 | amyloid + biopsy |  |  |  |  |  |  | NS | 66.9 (11.2) |  |  | 16 | 1 |
| Sueyoshi et al^13^ | 111 | 54 | CTS | 18 |  |  |  |  |  | 56/55 | 70.4 (9.1) | CTR | FTS | 18 |  |
|  |  |  |  | 36 |  |  |  |  |  |  | 59.5 (10.2) |  |  |  |  |
|  |  | 21 | rotator cuff tear |  |  |  |  | 5 |  |  | 71.9 (7.6) | rotator cuff repair | RCT | 5 |  |
|  |  |  |  |  |  |  |  | 16 |  |  | 55.5 (16) |  |  |  |  |
|  |  | 36 | LSS |  |  |  | 16 |  |  |  | 65.8 (6.3) | lumbar decompression | LF | 16 |  |
|  |  |  |  |  |  |  | 20 |  |  |  | 58.8 (15.4) |  |  |  |  |
| Takanashi et al^25^ | 232 | 232 | total population |  |  | 232 |  |  |  | 31/201 | 73 (9.1) | TKA | synovium from both joint sides |  |  |
|  |  | 211 | amyloid negative |  |  |  |  |  |  | 26/185 | 72.4 (9.3) |  |  |  |  |
|  |  | 21 | amyloid positive |  |  |  |  |  |  | 5/16 | 79 (4.6) |  |  | 21 |  |
| Uchihara et al^41^ | 25 | 10 | no family history of amyloidosis |  |  |  |  |  |  | 4/6 | NS (16-78) | AKA | periarticular F&A tissue | 3 |  |
|  |  | 15 |  |  |  |  |  |  |  | NS | NS (19-52) |  |  |  |  |
| Westermark et al^20^ | 26 | 5 | no amyloid |  |  |  | 26 |  |  | 2/3 | 57.8 (9.9) | lumbar decompression | LF, bone fragments, other CT |  |  |
|  |  | 21 | amyloid + |  |  |  |  |  |  | 11/10 | 68.1 (44-87) |  |  |  |  |
|  |  | 5 | TTR amyloid + |  |  |  |  |  |  | 3/2 | 79 (5.6) |  |  | 5 |  |
| Yanagisawa et al^21^ | 75 (116 samples) | 43 | LSS with amyloid |  |  |  | 95 |  |  | 26/17 | 74 (7.6) | lumbar decompression | LF | 43 |  |
|  |  | 52 | LSS no amyloid |  |  |  |  |  |  | 42/10 | 68 (6.4) |  |  |  |  |
|  |  | 12 | LDH no amyloid |  |  |  |  |  |  | 10/2 | 41.1 (10.6) | discectomy |  |  |  |
|  |  | 9 | LDH with amyloid |  |  |  |  |  |  | 7/2 | 57.6 (10.1) |  |  |  |  |
| Yanagisawa et al^26^ | 52 | 18 | meniscus |  |  | 52 |  |  |  | 7/11 | 79.1 (5.3) | TKA | meniscus, cartilage, synovium | 18 |  |
|  |  | 8 | articular cartilage |  |  |  |  |  |  | 3/5 | 79.3 (5.1) |  |  | 8 |  |
|  |  | 6 | synovial membrane |  |  |  |  |  |  | 1/5 | 78.7 (5.4) |  |  | 6 |  |
| Zegri-Reiriz et al^55^ | 101 | 101 | total population | 101 |  |  |  |  |  | 32/69 | 69 | cardiac evaluation for cardiomyopathy | no biopsy | | |
|  |  | 2 | ATTRwt |  |  |  |  |  |  | 0/2 | 85.5 (85-86) |  |  |  |  |
|  |  | 1 | AL |  |  |  |  |  |  | 1/0 | 80 |  | myocardial |  | 1 |

Table 3. Patient demographics of included studies. AL (immunoglobulin light-chain amyloidosis); AKA (above knee amputation); ATTR (transthyretin amyloidosis); ATTRm (mutant transthyretin amyloidosis); ATTRwt (wild-type transthyretin amyloidosis); CT (connective tissue); CTR (carpal tunnel release); CTS (carpal tunnel syndrome); F&A (foot and ankle); FTS (flexor tenosynovium); HF (heart failure; Ig (immunoglobulin); LDH (lumbar disk herniation); LF (ligamentum flavum); LSS (lumbar spinal stenosis); - (negative); NS (not specified); + (positive); RCR (rotator cuff repair); RCT (rotator cuff tendon); TCL (transverse carpal ligament); THA (total hip arthroplasty); TKA (total knee arthroplasty); TTR (transthyretin); TTR V30M (methionine for valine codon 30 of transthyretin gene)
